# Comparing Two Inferior Oblique Weakening Procedures: Disinsertion versus Myectomy

**DOI:** 10.18502/jovr.v16i2.9085

**Published:** 2021-04-29

**Authors:** Kaveh Abri Aghdam, Reza Asadi, Mostafa Soltan Sanjari, Ali Sadeghi, Meshkat Razavi

**Affiliations:** Eye Research Center, The Five Senses Institute, Rassoul Akram Hospital, Iran University of Medical Sciences, Tehran, Iran

**Keywords:** Disinsertion of Inferior Oblique Muscle, Inferior Oblique Muscle Overaction, Strabismus

## Abstract

**Purpose:**

To compare two methods for treating inferior oblique overaction (IOOA): disinsertion versus myectomy of the muscle.

**Methods:**

In this prospective interventional case series, patients were randomly assigned to undergo either IO myectomy or disinsertion. The changes in vertical and horizontal deviations following these two surgical procedures were evaluated. The postoperative IO function of grade 0 or +1 and the fundus extorsion of grade 0 or +1 was considered as the successful outcome.

**Results:**

Thirty-six patients (50 eyes) with a mean age of 12.67 ± 4.05 years were included. In the myectomy group, the mean preoperative hyperdeviation in adduction was 29.5 ± 9.32 prism diopter (PD), which decreased to 9.15 ± 7.86 PD after surgery (*P* = 0.001). In the disinsertion group, these measurements were 32.73 ± 12.42 and 12.65 ± 9.34 PD before and after the surgery, respectively (*P* = 0.001). The success rate of surgery based on the IOOA grading was 87.4% and 92.3% in the myectomy and disinsertion groups, respectively (*P* = 0.780). The successful correction rate of abnormal fundus torsion was 91.6% in the myectomy and 88.4% in the disinsertion group (*P* = 0.821). In comparison, 48% of the cases in the myectomy group and 50% in the disinsertion group were within the normal range of torsional position postoperatively (*P* = 0.786). There was no statistically significant difference in terms of changes in the horizontal or vertical deviations, V-pattern, and dissociated vertical deviation between the two groups.

**Conclusion:**

Both surgical techniques seem to be effective for treatment of inferior oblique muscle overaction.

##  INTRODUCTION

Of the esotropic and the exotropic patients, 70% and 30% have inferior oblique overaction (IOOA) respectively. Oblique muscle dysfunction is the primary cause of pattern strabismus. The most common type of pattern strabismus is V pattern esotropia with IOOA.^[1]^ Inferior oblique (IO) weakening procedures are self-adjusting; different amounts of deviation can be corrected surgically with the same method.^[2]^

Surgical procedures, including myectomy, myotomy, recession, total extirpation, disinsertion, denervation, and IO muscle fixation, can successfully weaken the IO muscle. Seemingly, none of them is superior to the other methods. Surgeon's preference and skill are the main factors in decision-making about the type of procedure. IO disinsertion is a safe and straightforward procedure without a need for suturing. However, the IO recession is preferred to disinsertion, myectomy, and myotomy by many surgeons because of the ability to achieve a graded response^[3]^ and the lower risk of unpredictable reattachment of IO muscle to the sclera.^[4]^ Some studies have supported IO disinsertion because of its simplicity, decreased risk of hemorrhage, and a similar success rate to other methods.^[5,6,7,8]^ In contrast, some authors believe that IO myectomy is more effective, despite the increased risk of bleeding using this surgical technique. ^[9]^

Considering the controversial opinions on the effectiveness of these surgical procedures, we conducted this study to evaluate the outcomes of IO disinsertion versus myectomy.

##  METHODS

This prospective interventional case series was conducted on 50 eyes of 36 patients from August 2016 to September 2017 at Rassoul Akram Hospital, Tehran, Iran. The Ethics Committee of Iran University of Medical Sciences approved the study design, and the study was conducted in compliance with the Declaration of Helsinki; written informed consent was obtained from all patients or their parents before the start of the protocol.

**Figure 1:**
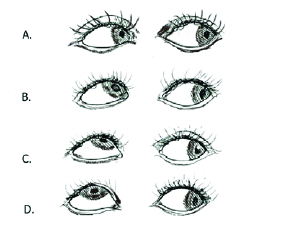
Schematic picture for classification of inferior oblique overaction (IOOA): (A) Grade +1: minimal upshoot of the adducting eye. (B) Grade +2: obvious upshoot of the adducting eye when the abducting eye looks straight across at the lateral canthus. (C) Grade +3: severe upshoot of the adducting eye is seen with the abducting eye in straight abduction. (D) Grade 4: very severe upshoot is considered as grade +4 of IOOA.

For grading of IOOA, if the abducting eye was the fixing eye and was fixating straight in abduction, minimal upshoot of the adducting eye was considered as grade +1 of IOOA. The adducting eye's obvious upshoot when the abducting eye looks straight across at the lateral canthus was rated as grade +2 of IOOA. The severe upshoot of the adducting eye was recognized as +3 of IOOA. A very severe upshoot of the adducting eye was considered as +4 of IOOA.^[10]^ Figure 1 shows the IOOA grading schematically.

All patients with IOOA ≥ +2 requiring IO muscle-weakening operation were included in this study. All participants underwent a comprehensive ophthalmologic examination, and those with prior strabismus surgery, restrictive strabismus, a history of neurologic, genetic, or craniofacial disorders, and simultaneous dissociated vertical deviation (DVD) were excluded. Fundus photography and indirect ophthalmoscopy were used for the assessment of ocular torsion before and after the surgery. The amount of abnormal torsion was determined using a grading system from “trace" to “+4," as described by Guyton.^[10]^ Normally, the fovea is within the upper third of the optic disc in the indirect ophthalmoscope view. For each one-eighth of the disc diameter that fovea lies upper than this border, +1 fundus extorsion is estimated.^[11]^

The prism and alternate cover test were used to measure the vertical and horizontal deviations before the surgery compared to final values. The deviation in the primary position at both near and far fixation and lateral gaze at distance fixation was recorded. V pattern existed when the difference between the horizontal deviation at the upper 30 degrees and lower 30 degrees was ≥15 prism diopters (PDs). IOOA in a patient with a positive head tilt test was classified as secondary IOOA.

Each patient was randomly assigned to the myectomy or disinsertion groups. In bilateral cases, both eyes underwent the same surgical procedure, and the operations were performed by one surgeon (MSS). The successful outcome was defined as the final IO function of grade +1 or 0 and the fundus extorsion of grade 0 or +1.

### Surgical Technique

All operations were performed under general anesthesia. In both groups, a small fornix-based incision was developed inferotemporally, and Tenon's capsule incision was made to reach the bare sclera. The lateral rectus muscle was secured on a muscle hook. IO muscle was dissected, isolated from surrounding tissues, and then the hook holding the lateral rectus was removed. In the disinsertion group, the muscle was disinserted and released to retract into the Tenon's sleeve. In the myectomy group, two muscle clamps 5-mm apart from each other were used to hold the muscle, and the muscle located between two clamps was excised and removed. Then the cut ends were cauterized. The muscle was released, and the proximal portion was allowed to be pulled back into the Tenon's sleeve. To ensure that no residual fiber remained, Guyton's exaggerated forced duction test was performed in both groups.^[12]^ Horizontal eye muscle surgery was performed based on the type of horizontal deviation after IO weakening.

Patients were evaluated one day, one week, one month, and six months following the surgery to determine the grade of IOOA, fundus torsion, horizontal or vertical deviation, V pattern, and DVD. An experienced orthoptist who was unaware of the type of surgery performed the measurements in all patients.

### Statistical Analysis

Statistical data analysis was performed with the SPSS 16.0 statistical software package (SPSS Inc., Chicago, IL, USA). Each eye was considered a single case to compare the IO muscles' function following myectomy and disinsertion. For evaluating the horizontal and vertical deviations, V pattern, or DVD, each patient was considered as one case for statistical evaluation. For describing data, mean ± standard deviation, frequency, and percentage were used. For evaluation of the difference between groups, the Mann–Whitney U-test, the Wilcoxon signed-rank test, Chi-square, and Fisher exact tests were conducted. *P*-values < 0.05 were considered statistically significant.

##  RESULTS

Overall, 36 patients (50 eyes) were enrolled in this study. The patients' mean age was 12.67 ± 4.05 years (range, 6 to 22 years), and 52% of them were male. The mean follow-up time was 6.45 ± 4.3 and 7.03 ± 4.8 months in the myectomy and disinsertion groups, respectively (*P* = 0.359). Fourteen patients had bilateral IOOA (six patients in the myectomy and eight patients in the disinsertion groups); the same technique was used in bilateral cases. Secondary IOOA (ipsilateral superior oblique underaction) was found in 16 eyes, including 29.1% of the eyes in the myectomy group and 34.6% in the disinsertion group. Table 1 represents the baseline demographic and clinical characteristics of the patients. They were matched in terms of age, sex, amount of vertical deviation, and the differences between groups were not statistically significant.

**Table 1:** Baseline demographic and clinical characteristics of the patients.


**Variable**	**Myectomy group**	**Disinsertion group**	**** ***P*** **-value**
Patient number	(18 patients, 24 eyes)	(18 patients, 26 eyes)	
Age (yr)	13.47 ± 4.62	11.96 ± 3.39	0.938
Sex (male/female)	10 (55.5%)/8 (44.4%)	9 (50%)/9 (50%)	0.605
Mean follow-up (month)	6.45 ± 4.3	7.03 ± 4.8	0.359
Unilateral/Bilateral	12 (66.6%)/6 (33.38%)	10 (55.5%)/8 (44.4%)	0.781
BCVA (LogMAR)	0.22 ± 0.14	0.23 ± 0.17	0.838
Spherical equivalent	+2.6 ± 3.01	+3 ± 3.24	0.515
Primary/Secondary IOOA	17 (70.8%)/7 (29.2%)	17 (65.3%)/9 (34.7%)	0.573
BCVA, best-corrected visual acuity; LogMAR, logarithm of the minimum angle of resolution; IOOA, inferior oblique muscle overaction			

**Table 2:** Distribution of eyes with various grades of IOOA and fundus torsion in each group pre- and postoperatively


	**Preoperative**		**Postoperative**	
	**Myectomy (24 eyes)**	**Disinsertion (26 eyes)**	**Myectomy (24 eyes)**	**Disinsertion (26 eyes)**
Grade of IOOA		
+4	5 (20.8%)	6 (21.13%)	0	0
+3	10 (41.6%)	13 (50%)	0	0
+2	9 (37.5%)	7 (26.9%)	3 (12.5%)	2 (7.6%)
+1	0	0	10 (41.6%)	5 (19.2%)
0 or trace	0	0	11 (45.8%)	19 (73%)
Grade of fundus torsion		
+4	7 (29.1%)	6 (23.07%)	0	0
+3	11 (45.8%)	10 (38.4%)	0	0
+2	6 (25%)	10 (38.4%)	2	3 (11.5%)
+1	0	0	11 (45.8%)	10 (38.4%)
0 or trace	0	0	11 (45.8)	13 (50%)
IOOA, inferior oblique overaction				

**Table 3:** Amount of hypertropia in the primary position and adduction before and after the surgery in the unilateral cases


**Myectomy**				**Disinsertion**		
	**Before**	**After**	**** ***P*** **-value**	**Before**	**After**	**** ***P*** **-value**
HT in PP (PD)	12.7 ± 3.9	3.8 ± 2.6	0.001	13.42 ± 2.8	4.06 ± 2.7	0.001
HT in Add (PD)	29.5 ± 9.32	9.15 ± 7.86	0.001	32.73 ± 12.42	12.65 ± 9.34	0.001
Add, adduction; HT, hypertropia; PD, prism diopters; PP, primary position						

**Table 4:** Comparison of myectomy and disinsertion in the subgroups of primary and secondary IOOA


	**Myectomy**	**Disinsertion**	**** ***P*** **-value**
Primary IOOA	17	17	0.999
Secondary IOOA	7	9	0.742
Mean reduction in HT in Add (primary IOOA) (PD)	20.86 ± 11.45	21.46 ± 12.76	0.864
Mean reduction in HT in Add (secondary IOOA) (PD)	21.43 ± 11.38	22.42 ± 12.19	0.832
Success rate based on IOOA grading (primary IOOA)	88.2%	88.2%	0.999
Success rate based on grading of fundus torsion (primary IOOA)	85.7%	100%	0.853
Success rate based on the IOOA grading (secondary IOOA)	88.2%	88.2%	0.999
Success rate based on the grading of fundus torsion (secondary IOOA)	100%	85.7%	0.853
Add, adduction; HT, hypertropia; IOOA, inferior oblique overaction; PD, prism diopters; PP, primary position			

Twenty-seven patients had concurrent horizontal deviations in the primary position (esotropia in 15 patients and exotropia in 12 cases) and received simultaneous surgery on the horizontal muscles during IO weakening surgery. Nine patients underwent IO weakening alone. No V pattern was observed postoperatively. Table 2 demonstrates the grades of IOOA and fundus torsion in both groups before and after the surgery. The distribution of IOOA was similar between the groups preoperatively (*P* = 0.867). At baseline, 62.5% of the eyes in the myectomy group and 73.1% in the disinsertion group had +3 or +4 IOOA. The successful outcome was defined as the final IO function of grade +1 or 0 and the fundus extorsion of grade 0 or +1. The success rate of surgery based on IOOA grading was 87.4% and 92.3% in the myectomy and disinsertion groups, respectively (*P* = 0.780). The successful correction rate of abnormal fundus torsion was 91.6% in the myectomy group and 88.4% in the disinsertion group (*P* = 0.821). The median preoperative grade of IOOA was +3 (with an interquartile range of +1) in both groups (*P* = 0.084). The median postoperative grade of IOOA was +1 (with an interquartile range of +1) in both groups (*P* = 0.097). The median preoperative grade of fundus torsion was +3 (with an interquartile range of +2) in the myectomy group and +3 (with an interquartile range of +1) in the disinsertion group (*P* = 0.642). The median postoperative grade of fundus torsion was +1 (with an interquartile range of +1) in both groups (*P* = 0.960).

Table 3 shows the amount of hypertropia in the primary position and adduction before and after the surgery in both groups. The amount of hypertropia in the primary position and adduction significantly decreased in both groups (*P* = 0.001). There was no statistically significant difference between the two groups in terms of the mean reduction in hypertropia in the primary position (*P* = 0.086) and adduction (*P* = 0.187). Table 4 demonstrates the comparison between the myectomy and disinsertion within two subgroups of primary and secondary IOOA. No significant difference was found between the two subgroups regarding the mean reduction in hypertropia in the primary position, adduction, success rate based on IOOA, and fundus torsion (all *P*-values > 0.05).

Postoperative IO underaction was observed only in one case in the myectomy group. In one single case in the disinsertion group, IOOA developed in the opposite eye. No new DVD was developed postoperatively. Significant complications such as scleral perforation, retrobulbar hemorrhage, or fat adherence syndrome did not occur during the study period.

##  DISCUSSION 

According to our results, both myectomy and IO disinsertion techniques can improve IOOA and achieve gratifying results. Disinsertion of IO muscle is a safe and effective treatment for most cases with IOOA. Jones et al performed IO disinsertion on 337 patients and found a successful result in 88% of primary IO disinsertion.^[5]^ For secondary IOOA, a successful result was reported in 72% of patients. Similarly, Duranglu^[2]^ reported a success rate of 73.6% in the disinsertion group of patients with infantile esotropia and primary IOOA.^[13]^ Our findings are also in line with previous findings of Sanjari et al, who compared three surgical procedures for the treatment of IOOA, including disinsertion, myectomy, and anterior transposition.^[14]^ They concluded that all these procedures were useful for treating primary and secondary IOOA with minimal side effects.

One of the most common phenomena following unilateral IO weakening procedures is the occurrence of IOOA in the contralateral eye. The reported average time from the first disinsertion to the contralateral second disinsertion was about 23 months.^[5]^ In our study, IOOA in the opposite eye occurred only in only one eye in the disinsertion group. However, the length of follow-up is not enough to conclude. De Angelis et al have observed multiple insertions and duplications of IO muscles during surgery in 17% and 8% of cases, respectively.^[12]^ They concluded that the recurrence of IOOA following the weakening surgery might be due to incomplete muscle capture in the surgical procedure; however, we performed Guyton's exaggerated forced duction test for the IO muscles at the end of surgery for all patients.^[15]^

Parks compared IO muscle recession with disinsertion and myectomy.^[16]^ He found a recession to be superior due to the occurrence of an adherence syndrome following some cases of disinsertion and myectomy. However, recession and myectomy are more complicated than disinsertion, and there is no logical reason for the development of adherent syndrome. We observed fat adherence syndrome neither in the myectomy nor in the disinsertion groups.

In our study, the amount of preoperative hypertropia in the primary position and adduction was similar in both groups; therefore, the similar corrective effect of both surgical methods could be due to the same effect on the function of the inferior rectus muscle. Wertz et al^[2]^ studied the muscle length shortening following IO myectomy and disinsertion surgery in Rhesus monkeys. They observed a wide variation in the IO reattachment site. Therefore, the same weakening procedure for different amounts of IOOA could result in similar outcomes, which might be related to the self-adjusting nature of IO myectomy and disinsertion. They also found that the average muscle length was equal in both procedures after 6 to 20 weeks. IO muscle shortened to two-thirds of its normal length. This similar effect on muscle length could also be the reason for a similar effect on muscle function and comparable postoperative outcomes.

Awadein and Gawdat reported that symmetric IO myectomy might have a symmetrizing effect in cases of asymmetric IOOA.^[17]^ They measured the degree of IOOA and the degree of fundus torsion by Guyton's method and reported that the degree of fundus torsion in their cases did not always correlate with the degree of IOOA; this finding may also relate to the self-adjusting nature of IO muscle, which allows muscle retraction to find a proximal site of insertion. The degree of IO tightness partially determines the new insertion site. The tightness of IO muscle on the side with more overaction would cause more retraction and gain more proximal new insertion.

Akbari et al showed that IO disinsertion effectively corrected primary position vertical deviation of unilateral congenital superior oblique palsy patients as IO myectomy when preoperative vertical deviation was ≤15D.^[18]^ However, when preoperative hypertropia was >15D, IO myectomy had a more prominent effect in reducing primary position vertical deviation. However, the nonrandomized design of their study may confound these results.

Finally, Chang et al reviewed four different IO muscle-weakening surgeries for vertical strabismus in superior oblique palsy: myectomy, recession, anterior transposition, and disinsertion.^[19]^ They could not find high-quality evidence to support recommendations for surgical treatment choice in patients with vertical strabismus due to superior oblique palsy.

Nevertheless, this study showed that both IO myectomy and IO disinsertion are effective and safe treatments for IOOA, and both techniques have similar successful results. Disinsertion is a safe procedure eliminating clinically significant V pattern and high-grade extorsion, and it is not associated with any clinically significant under or overaction according to our results. Disinsertion is also technically easier than myectomy with less bleeding, and IO muscle is preserved for potential future surgeries if needed.

The present study has several limitations, including a small sample size in each group, short follow-up time, and subjective measurement of fundus torsion. A randomized controlled trial with a large sample size and longer follow-up could lead to more accurate results.

In conclusion, both disinsertion and myectomy can effectively reduce the degree of IOOA, and the success rate is comparable in the two surgical methods.

##  Financial Support and Sponsorship

Nil.

##  Conflicts of Interest

The authors do not have any conflicts of interest.
